# Osthole stimulates bone formation, drives vascularization and retards adipogenesis to alleviate alcohol‐induced osteonecrosis of the femoral head

**DOI:** 10.1111/jcmm.15103

**Published:** 2020-03-05

**Authors:** Hongping Yu, Daoyu Zhu, Pei Liu, Qianhao Yang, Junjie Gao, Yigang Huang, Yixuan Chen, Youshui Gao, Changqing Zhang

**Affiliations:** ^1^ Department of Orthopedic Surgery Shanghai Jiao Tong University Affiliated Sixth People's Hospital Shanghai China

**Keywords:** ethanol, osteonecrosis of the femoral head, osthole, vascularization

## Abstract

Characteristic pathological changes in osteonecrosis of the femoral head (ONFH) include reduced osteogenic differentiation of bone mesenchymal stem cells (BMSCs), impaired osseous circulation and increased intramedullary adipocytes deposition. Osthole is a bioactive derivative from *coumarin* with a wide range of pharmacotherapeutic effects. The aim of this study was to unveil the potential protective role of osthole in alcohol‐induced ONFH. In vitro, ethanol (50 mmol/L) remarkably decreased the proliferation and osteogenic differentiation of BMSCs and impaired the proliferation and tube formation capacity of human umbilical vein endothelial cell (HUVECs), whereas it substantially promoted the adipogenic differentiation of BMSCs. However, osthole could reverse the effects of ethanol on osteogenesis *via* modulating Wnt/β‐catenin pathway, stimulate vasculogenesis and counteract adipogenesis. In vivo, the protective role of osthole was confirmed in the well‐constructed rat model of ethanol‐induced ONFH, demonstrated by a cascade of radiographical and pathological investigations including micro‐CT scanning, haematoxylin‐eosin staining, TdT‐mediated dUTP nick end labelling, immunohistochemical staining and fluorochrome labelling. Taken together, for the first time, osthole was demonstrated to rescue the ethanol‐induced ONFH *via* promoting bone formation, driving vascularization and retarding adipogenesis.

## 1 INTRODUCTION

Osteonecrosis of the femoral head (ONFH) is a devastating disease which largely advances to femoral head collapse as the natural history and finally necessitates total hip arthroplasty.^1^, ^2^ Although the exact pathogenesis of non‐traumatic ONFH has not been fully elucidated, several risk factors have been identified, including corticosteroids medication, alcohol consumption and several autoimmune diseases.^1^ Actually, alcoholism is one of the leading risk factors for ONFH worldwide,^3^, ^4^, ^5^ and epidemiologic studies indicated that 20%‐45% of ONFH patients were due to alcohol overuse.^6^

Bone mesenchymal stem cells (BMSCs) are predominant precursor cells for bone regeneration and remodelling.^7^, ^8^ Accumulating evidence indicates that alcohol impairs bone homoeostasis.^9^ Alcohol inhibits BMSCs differentiation into osteoblast; on the contrary, it promotes adipogenesis of BMSCs.^10^, ^11^ Additionally, alcohol critically restrains DNA synthesis and proliferation of osteoprogenitor cells.^12^ Moreover, high concentration of alcohol has a detrimental effect on neovascularization.^13^, ^14^ Therefore, alcohol may cause ONFH via inhibiting osteogenic activity of BMSCs directly and impairing vascularization to alter bone homoeostasis indirectly.

Recently, traditional Chinese medicine (TCM) is used to tackle bone diseases with increasing interest. Previous studies have found several herbs, and their monomers could ameliorate alcohol‐induced ONFH in animal models.^15^, ^16^ Osthole, a *coumarin* bioactive derivative, obtained from many medical plants, is commonly used as ingredients in herbal medicine and functional foods.^17^, ^18^ With the in‐depth investigations, osthole is revealed to possess a wide range of different pharmacological effects, including hepatoprotective, vasorelaxant, antileishmanial, neuroprotective, spasmolytic and antimicrobial properties.^19^, ^20^, ^21^, ^22^, ^23^, ^24^, ^25^, ^26^ Notably, it was demonstrated that osthole significantly promoted the proliferation of osteoblast‐like cells.^27^ Extracellular signal‐regulated kinase and β‐catenin/BMP signalling pathway might be involved in the osteogenic differentiation stimulated by osthole.^28^, ^29^ Intriguingly, osthole could be as effective as 17β‐estradiol in rescuing bone loss in ovariectomy‐induced osteoporosis in rats.^30^ Moreover, osthole could drive fracture healing *via* promoting endochondral ossification.^31^ The beneficial effects of osthole imply that it might yield protective results for alcohol‐induced ONFH. Herein, we demonstrated that osthole could stimulate bone formation, drive vascularization and retard adipogenesis, thus to alleviate alcohol‐induced ONFH in the rat model.

## 2 MATERIALS AND METHODS

### 2.1 Cell culture

Bone mesenchymal stem cells were obtained from the femurs and tibias of 4‐week‐old Sprague‐Dawley (SD) rats according to the previous method.^32^ BMSCs were cultured in α minimum essential medium (α‐MEM) (Gibco) with 10% foetal bovine serum (FBS) (Invitrogen), 1% penicillin/streptomycin (Invitrogen) at 37°C in a humidified atmosphere of 5% CO_2._ The BMSCs used in all experiments were between three and seven passages. Human umbilical vein endothelial cells (HUVECs) were purchased from Procell and cultured in endothelial cell medium (ECM) (Gibco).

### 2.2 Flow cytometry

Flow cytometry was used to test the surface markers of mesenchymal stem cells at passage three. In brief, adherent cells were washed with phosphate buffered saline (PBS) and disassociated by 0.05% trypsin (Gibco) and centrifuged at 350 g for 5 min. Next, the cells were rinsed twice with PBS and blocked with 1% FBS for 30 min at 4°C. Then, cells were incubated with fluorescein isothiocyanate (FITC)‐conjugated monoclonal antibodies for rat CD29, CD90, CD105, CD31, CD34 and CD45 (Proteintech) for 1 hour at 4°C. The same amounts of cells without incubating any antibodies were used as the negative control. Finally, the cells were washed with PBS three times and analysed by flow cytometry.

### 2.3 Multilineage differentiation of BMSCs

The multilineage differentiation capacity of BMSCs was detected according to the previous methods.^16^ In brief, 2 × 10^5^ of BMSCs were seeded in 6‐well plates with α‐MEM. After 80% confluence, osteogenic medium (Cyagen) was used to induce osteogenic differentiation for 21 days, and the mineralized nodules were stained by Alizarin red. Adipogenic medium (Cyagen) was used to induce adipogenic differentiation for 21 days, and the lipid vacuoles were stained by oil red O. Chondrogenic medium (Cyagen) was used to induce chondrogenic differentiation for 28 days, and the frozen sections of pellets were stained by Alcian blue.

### 2.4 Cell proliferation

The effects of ethanol (50 mmol/L) and osthole (10, 50, 100 μmol/L) on BMSCs or HUVECs proliferation were tested by Cell Counting Kit‐8 assay (Beyotime). The dose of ethanol was based on the previous work.^10^ Briefly, cells were seeded in 96‐well plates at 5 × 10^3^ per well in 100 μL culture medium. To measure cell proliferation, 10 μL CCK‐8 solution and 90 μL medium were added to each well, and then, the plates were incubated in 37°C for 2 hours. The absorbance values of supernatants at 450 nm were measured.

### 2.5 Mineralization assay

In order to detect the roles of ethanol, osthole and Wnt/β‐catenin pathway in osteogenic differentiation, BMSCs were incubated with ethanol (50 mmol/L), different doses of osthole (10, 50, 100 μmol/L) and JW74 (1 μmol/L; MCE, Monmouth Junction, NJ) for specific time. Briefly, 2 × 10^5^ of BMSCs were seeded in 6‐well plates with α‐MEM. After 80% confluence, osteogenic medium (Cyagen) was used to induce osteogenic differentiation,^33^ and the medium was refreshed every two days. Alizarin red staining was performed at day 21. ALP activity, which was measured using a microplate test kit at 560 nm, as well as ALP staining, was obtained at day 7 and 14. Images were captured with a LEICA microscope.

### 2.6 Adipogenesis assay

To investigate the effects of ethanol and osthole on adipogenic differentiation, BMSCs were incubated with ethanol (50 mmol/L) and osthole (10, 50, 100 μmol/L) for 21 days. In brief, 2 × 10^5^ of BMSCs were seeded in 6‐well plates with α‐MEM. After 80% confluence, adipogenic medium (Cyagen) was used to induce adipogenic differentiation, and the medium was refreshed according to the manufacturer's protocol. Oil red O staining was performed at day 21, and images were captured with a LEICA microscope.

### 2.7 Tube formation assay

The effects of ethanol (50 mmol/L) and osthole (10, 50, 100 μmol/L) on vasculogenesis were performed by the tube formation assay. Briefly, 50 μL/well Matrigel (BD Bioscience) was added to the 96‐well plate. Subsequently, 4 × 10^4^ HUVECs/well were seeded on the surface of Matrigel after gelatinization in 37°C for 30 min. The tube formation images were captured by a LEICA phase contrast microscope, and the number of complete capillaries and nodes of each hole were calculated.

### 2.8 Quantitative real‐time polymerase chain reaction (QPCR)

BMSCs and HUVECs were cultured in 6‐well plates in different dissolution for 48 hours, and then, total RNA was extracted from each hole according to the manufacturer's protocol (TaKaRa). Reverse transcriptase reactions contained the purified total RNA and 50 nmol/L RT primer. M‐MLV reverse transcriptase (TaKaRa) was used. QPCR was performed by a SYBR Premix Ex Taq protocol (TaKaRa) on an MX3005P system. GAPDH was used for the internal control gene of RNA expression. The primers were listed in Table S1.

### 2.9 Western blotting

Briefly, BMSCs and HUVECs were lysed with cell lysis buffer (50 mmol/L Tris‐HCl, 150 mmol/L NaCl, 1% Triton X‐100, 1% sodium deoxycholate, 0.1% SDS and 1 mmol/L PMSF). The protein concentrations were determined by the BCA assay reagent (Beyotime) according to the manufacturer's protocols. Equal amounts of proteins (30 μg) were electrophoresed on 10% SDS‐PAGE, and the bands were transferred to polyvinylidene fluoride (PVDF) membranes, which were blocked with 5% (w/v) milk in 0.05 mol/L Tris‐buffered saline (TBS) for 1 hour at room temperature and incubated at 4℃ overnight with the primary antibodies against Runx 2, GAPDH, β‐catenin, PPARγ (CST) in Tris‐buffered saline Tween‐20 (TBST). Then, the PVDF membranes were washed three times in TBST and incubated for 1 hour with an HRP‐conjugated second antibody at room temperature. Finally, the membranes were washed three times and reacted with the SuperSignal West Pico kit (Thermo Scientific, Waltham, MA). Signals were quantified using scanning densitometry.

### 2.10 ELISA

Human umbilical vein endothelial cells were cultured in 6‐well plates for 48 hours, and then, cells were lysed with cell lysis buffer according to the manufacturer's protocol (Beyotime). Then, the proteins were diluted for analysis of VEGF content by ELISA (Neobioscience). The absorbance values at 450 nm were measured and used to calculate the VEGF content according to the standard curve.

### 2.11 Immunofluorescent staining

Bone mesenchymal stem cells were seeded on gelatin‐coated 35 mm confocal dishes for 24 hours with basal medium. Then, BMSCs were incubated with condition medium containing ethanol (50 mmol/L) or additional osthole (50 μmol/L) for 48 hours. Later, cells were washed with PBS twice and fixed with 4% paraformaldehyde for 30 min, permeabilized by 0.5% Triton X‐100 in PBS for 10 min and incubated with primary antibodies of COL I and OCN (CST) for 1 hour at room temperature. Cells were washed twice with PBS and incubated with the Alexa Fluor™ 488 secondary antibodies. Subsequently, cell nucleus and skeletons were stained with DAPI and phalloidine.

### 2.12 Animal experiment

Thirty 8‐week‐old male SD rats were used for animal experiment with the approval from the Animal Research Committee at Shanghai Sixth People's Hospital. All rats were randomly and equally divided into three groups: (a) control group, (b) ethanol group and (c) ethanol + osthole group. All rats were allowed to adapt to the Lieber‐DeCarli liquid diet for one week. For the next 6 weeks, in ethanol group, rats had an ethanol‐containing Lieber‐DeCarli liquid diet, containing 8% (w/v) ethanol (36% of daily calories).^34^ The rats in the ethanol + osthole group had the same ethanol diet but were co‐treated with osthole (100 mg/kg/d) by intraperitoneal injection,^17^, ^18^ and the rats in control group had the normal Lieber‐DeCarli liquid diet without ethanol (the ethanol was displaced by maltodextrin, with the same amount of calories). All rats were free access to the diets which were refreshed every day. Fluorescence staining was performed to monitor dynamic bone formation and mineralization. In brief, 20 mg/kg tetracycline (Aladdin), 10 mg/kg calcein‐AM (Aladdin) and 30 mg/kg alizarin red S (Aladdin) were injected intraperitoneally at week 0, 2 and 4 during the experiment.

### 2.13 Micro‐CT scanning and analysis

Rats were killed under general anaesthesia at the end of the 6th week, and bilateral femoral heads were obtained and fixed in 4% paraformaldehyde. All samples were scanned with a 9‐micron voxel size micro‐CT scanner (Skyscan 1176). The acquisition conditions are 35 kV of energy and 220 mA of intensity. The images were managed by CTVol software and reconstructed. Parameters, including bone mineral density (BMD), trabecular thickness (Tb.Th), trabecular number (Tb.N) and trabecular bone volume fraction (BV/TV), were calculated from the reconstructed images.

### 2.14 Histological and immunohistochemical staining

After micro‐CT scanning, specimens were decalcified with 10% EDTA for one month and then embedded in paraffin. The specimens were cut into 5‐μm‐thick sections and stained with haematoxylin and eosin (HE). For immunohistochemical staining, sections were deparaffinized, antigen retrieved, blocked and incubated with primary antibodies of COL I and OCN (CST) and relevant biotinylated secondary antibodies. Finally, sections were stained with DAB and counterstained with haematoxylin.

### 2.15 Apoptosis assay

TdT‐mediated dUTP nick end labelling (TUNEL) was used to detect the apoptosis within the femoral head. After the sections were deparaffinized and antigen retrieved, the TUNEL staining was performed according to the manufacture's protocols (Beyotime). Images were captured by LEICA DM 400 microscope.

### 2.16 Statistical analysis

All data were presented as means ± standard error of the mean (SEM). The differences between groups were determined by Student's *t* test or one‐way ANOVA with Bonferroni correction in SPSS 18 (IBM). *P < .05, **P < .01 and ***P < .001 were considered to be of statistical significance.

## 3 RESULTS

### 3.1 Identification of primary BMSCs

Flow cytometry was used to determine the purity of BMSCs. The positive rates of markers including CD29, CD90 and CD105 were 99.6%, 98.9% and 99.5%, whereas the markers including CD31, CD45 and CD34 were negligible. Next, in order to detect the capacity of multilineage differentiation, BMSCs were used to differentiate into osteogenic, chondrogenic and adipogenic lineage under respective induction medium. Pellet assay indicated that BMSCs could differentiate into chondrocytes (Figure S1A), oil red O staining revealed the differentiation of adipocytes (Figure S1B), and Alizarin red staining confirmed the extracellular mineralization *via* osteogenesis (Figure S1C). Primary BMSCs were demonstrated by flow cytometry and multilineage differentiation, in combination with the behaviour of adherent proliferation.

### 3.2 Osthole rescued the ethanol‐induced inhibition on osteogenic differentiation of BMSCs via Wnt/β‐catenin pathway

**Figure 1:**
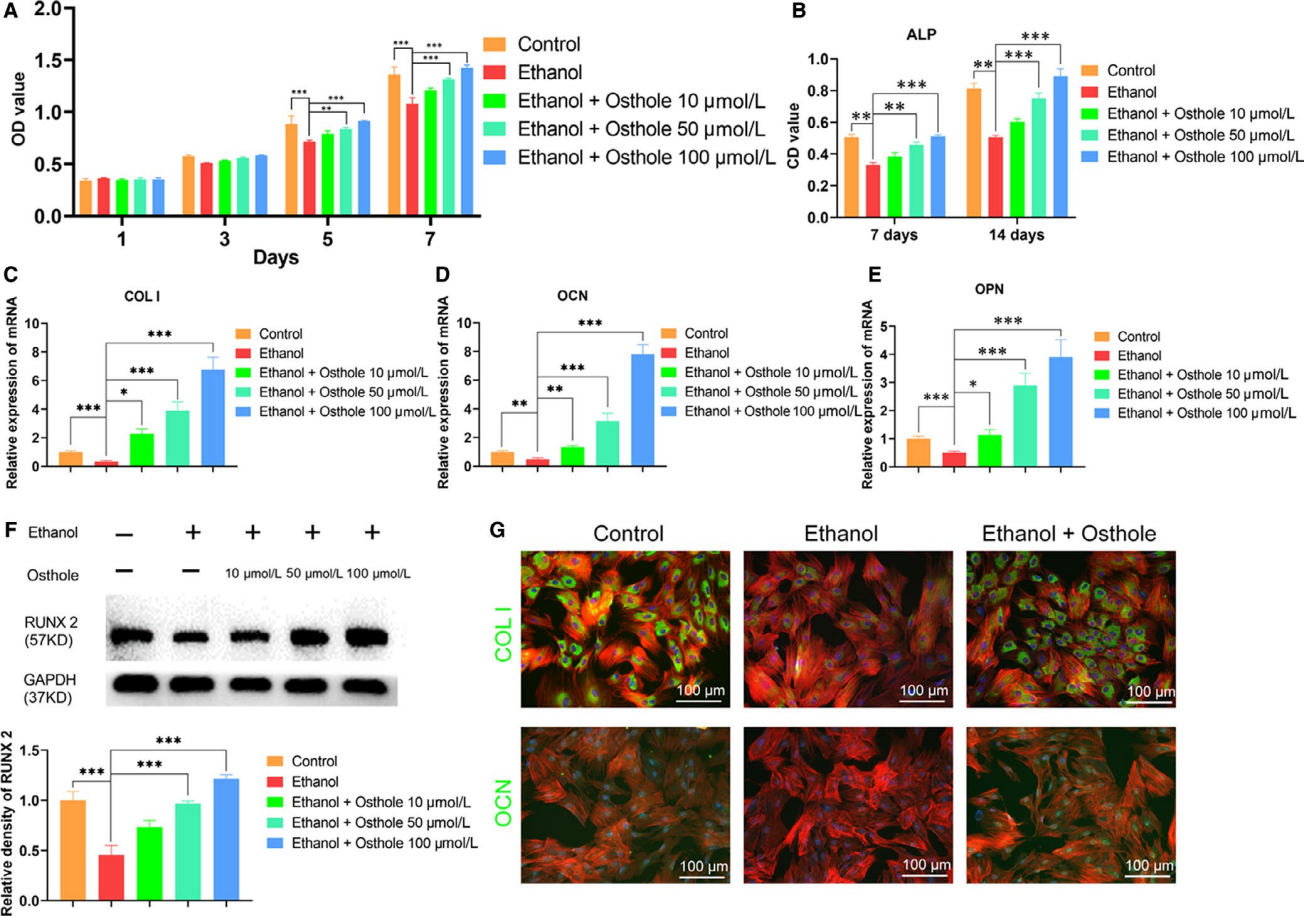
Osthole alleviated the ethanol‐induced inhibition on proliferation and osteogenesis of BMSCs. A, The proliferation of BMSCs incubated in medium supplemented with ethanol and osthole was determined by CCK 8 assay. Results were means ± SEM of four independent experiments in duplicate. B, The ALP activity of BMSCs cultured in osteogenic medium supplemented with ethanol and osthole was tested at day 7 and 14. Results were means ± SEM of four independent experiments in duplicate. C‐E, The mRNA level of COL I, OCN and OPN was decreased in BMSCs after 48 h incubation with ethanol but was substantially up‐regulated when treated with osthole. GAPDH was set as a normalization control. Results were means ± SEM of four independent experiments in triplicate. F, Runx2 was decreased by ethanol but was strengthened by osthole in BMSCs. Results were means ± SEM of four independent experiments. G, Immunofluorescence staining of COL I and OCN showed osthole reversed the anti‐osteogenic effect of ethanol in BMSCs. BMSCs were cultured for 48 h in Osteogenic medium supplemented with ethanol and osthole. Cytoskeletons were stained with phalloidine (red), and the nucleus was stained with DAPI (blue)

Firstly, CCK‐8 assay was employed to detect the proliferation of BMSCs. Ethanol (50 mmol/L) could significantly inhibit the proliferation of BMSCs at day 5 and 7. However, osthole (10, 50, 100 μmol/L) was able to rescue the inhibitory effect of ethanol in a dose‐dependent manner (Figure 1A).

**Figure 2:**
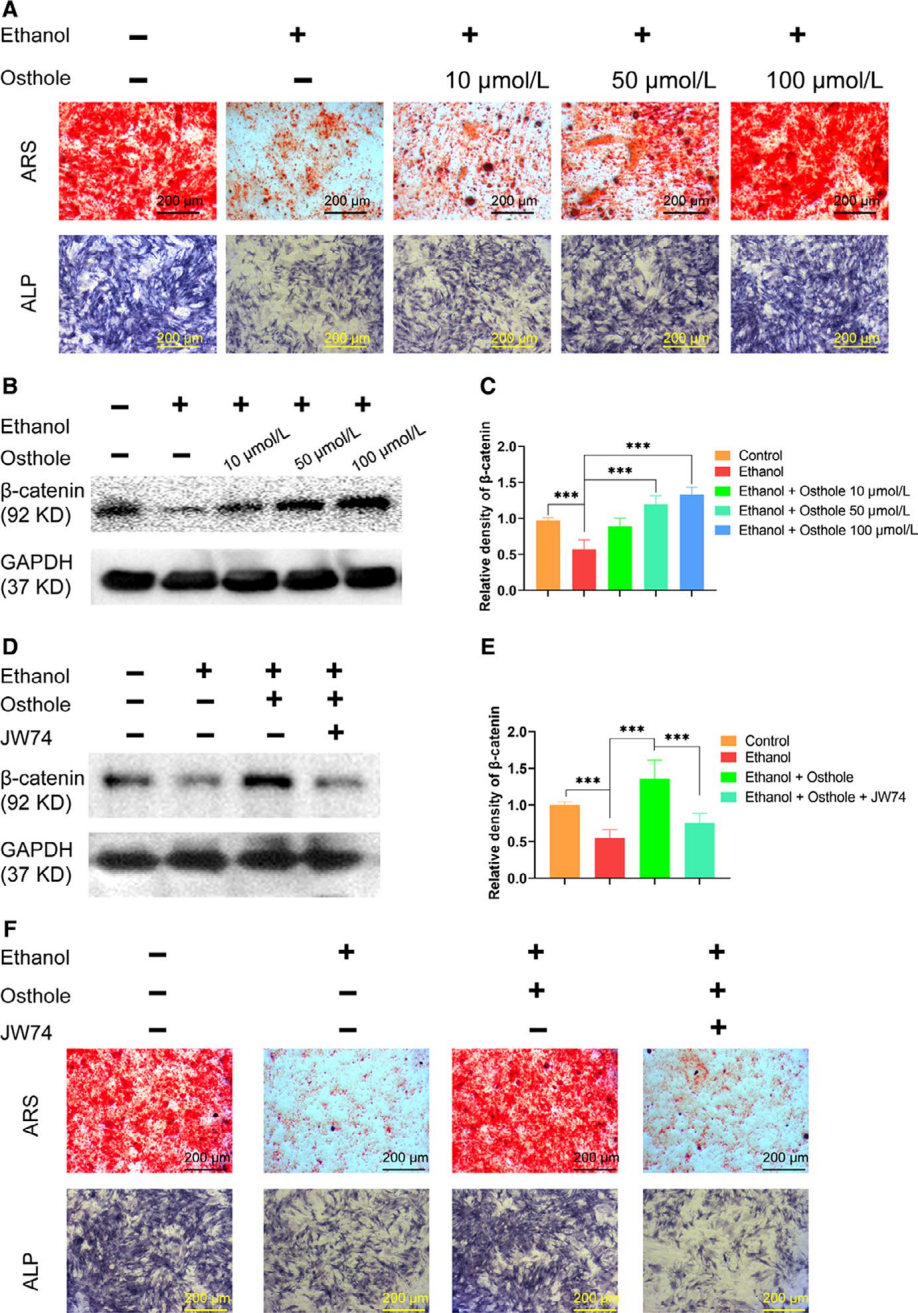
Osthole abolished the ethanol‐induced inhibition on mineralization *via* Wnt/β‐catenin‐dependent pathway. A, The Alizarin red and ALP staining of BMSCs incubated for 21 days in osteogenic medium with supplemented ethanol and osthole, showed the inhibition role of ethanol and the protection of osthole on extracellular mineralization. B,C, β‐catenin was decreased by ethanol and increased by osthole in BMSCs. Results were means ± SEM of four independent experiments. D,E, Selective Wnt/β‐catenin antagonist JW74 abolished the protective effects of osthole. Results were means ± SEM of four independent experiments. F, JW74 abolished the reverse effects of osthole on ethanol‐induced decreased mineralized nodules deposition in BMSCs, revealed by ARS and ALP staining

As shown in Figure 1B, ethanol markedly down‐regulated ALP activity at day 7 and 14, whereas supplementation of 50 and 100 μmol/L osthole dramatically abolished the ethanol‐induced inhibition on ALP activity. However, the effect of osthole at 10 μmol/L was not significant. In terms of the osteogenic‐associated gene expression, COL I, OCN and OPN were obviously decreased in ethanol group at 48 hours; however, osthole could dose‐dependently reverse the inhibition (Figure 1C‐E). We also detected osteogenic‐associated protein by Western blot. It was demonstrated that Runx2 was remarkably decreased by ethanol, but the inhibition was obviously antagonized by higher doses of osthole (50, 100 μmol/L) (Figure 1F). In order to directly visualize and quantify the changes of osteogenic‐associated protein in BMSCs, immunofluorescence staining of COL I and OCN was performed. The results indicated that ethanol significantly impaired the expression of COL I and OCN when BMSCs were incubated with ethanol for 48 hours, whereas osthole (50 μmol/L) could reverse the inhibition of ethanol on BMSCs (Figure 1G). Finally, Alizarin red staining and ALP staining were remarkably weakened for ethanol‐treated BMSCs. However, osthole could rescue inhibited osteogenesis by ethanol in a dose‐dependent manner (Figure 2A).

Western blot assay was performed to determine whether Wnt/β‐catenin signalling was involved in alcohol‐induced ONFH. Compared to the control group, β‐catenin was significantly decreased in BMSCs after ethanol treatment. However, osthole could dose‐dependently reverse the inhibition of ethanol on β‐catenin production in BMSCs (Figure 2B,C). Wnt antagonist JW74 was used to investigate the role of Wnt/β‐catenin signalling pathway in the osteoprotective function of osthole.^35^ As shown in Figure 2D and E, JW74 (1 μmol/L) significantly abolished the up‐regulation of β‐catenin when BMSCs were co‐treated with ethanol and osthole. The role of Wnt/β‐catenin pathway was further investigated by ARS and ALP staining. It was revealed that JW74 could dramatically abolish the beneficial effect of osthole on extracellular mineralization (Figure 2F). Taken together, these data indicated that osthole could alleviate ethanol‐induced anti‐osteogenic effect in BMSCs *via* Wnt/β‐catenin signalling pathway.

### 3.3 Osthole retarded the ethanol‐induced adipogenic differentiation of BMSCs

**Figure 3:**
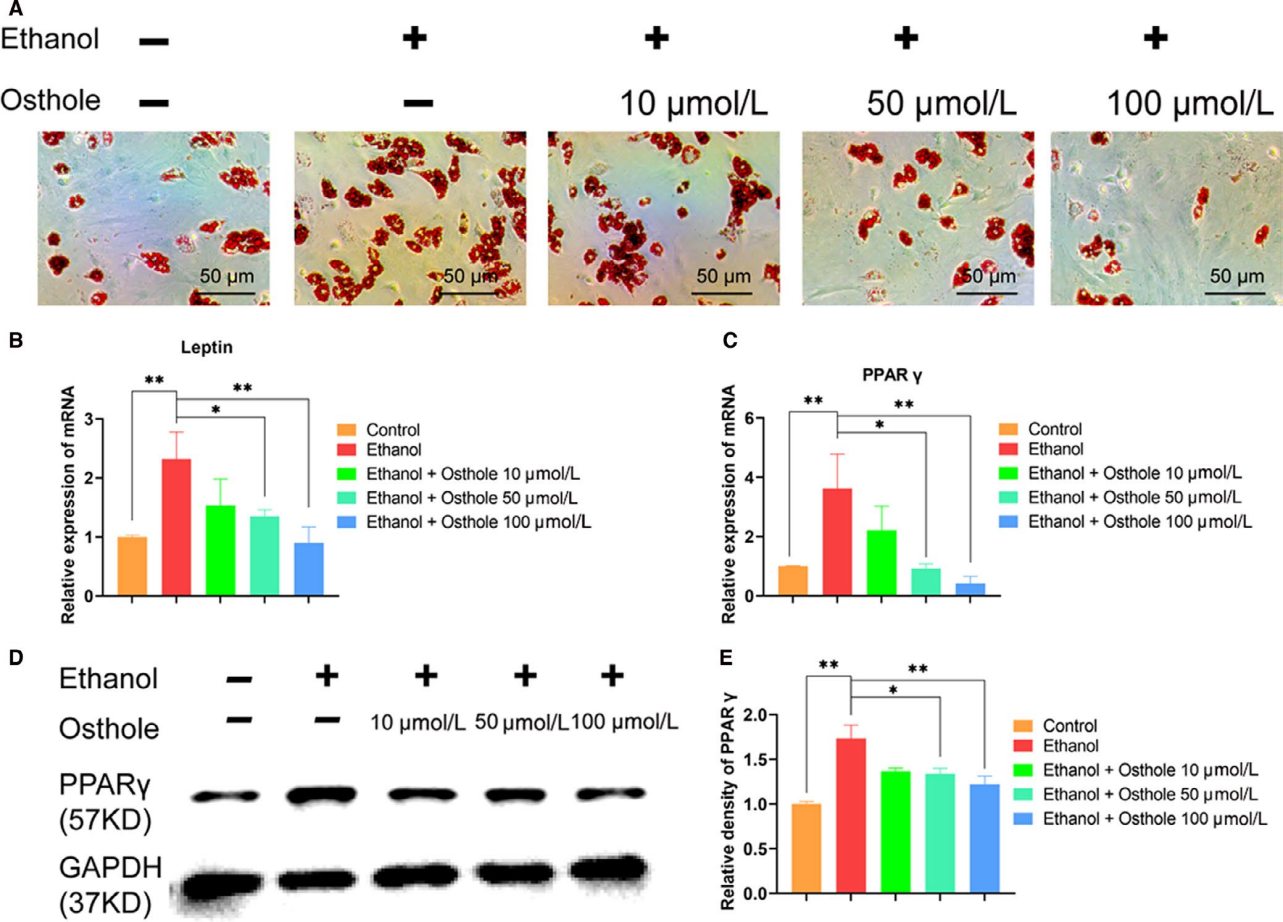
Osthole retarded the ethanol‐promoted adipogenic differentiation of BMSCs. A, The oil red O staining of BMSCs, which were incubated for 21 days with respective conditions, showed the effect of ethanol and osthole on adipogenesis. B,C, Leptin and PPARγ mRNA were substantially up‐regulated in BMSCs after 7 days incubation with ethanol; however, they were abolished when supplemented with osthole. Results were means ± SEM of four independent experiments in triplicate. D,E, The protein of PPARγ was increased by ethanol but was counteracted by additional osthole in BMSCs. Results were means ± SEM of four independent experiments

Adipogenic deposition is one of important pathological features in ONFH. Herein, oil red O staining was used to evaluate the role of ethanol and osthole in the adipogenic differentiation of BMSCs. As shown in Figure 3A, ethanol had a strong promotion of adipogenic activity of BMSCs, yielding a myriad of lipid vacuoles. However, osthole could dose‐dependently retard the promotion of adipogenesis by ethanol. Leptin and PPARγ, as pivotal adipokines, were markedly up‐regulated in ethanol group at day 7, but osthole could dose‐dependently counteract the stimulatory effect of ethanol in adipogenesis (Figure 3B,C). The protein level of PPARγ was also significantly up‐regulated by ethanol but was abolished by osthole in a dose‐dependent manner (Figure 3D,E).

### 3.4 Osthole rescued the ethanol‐induced inhibition of vascularization

**Figure 4:**
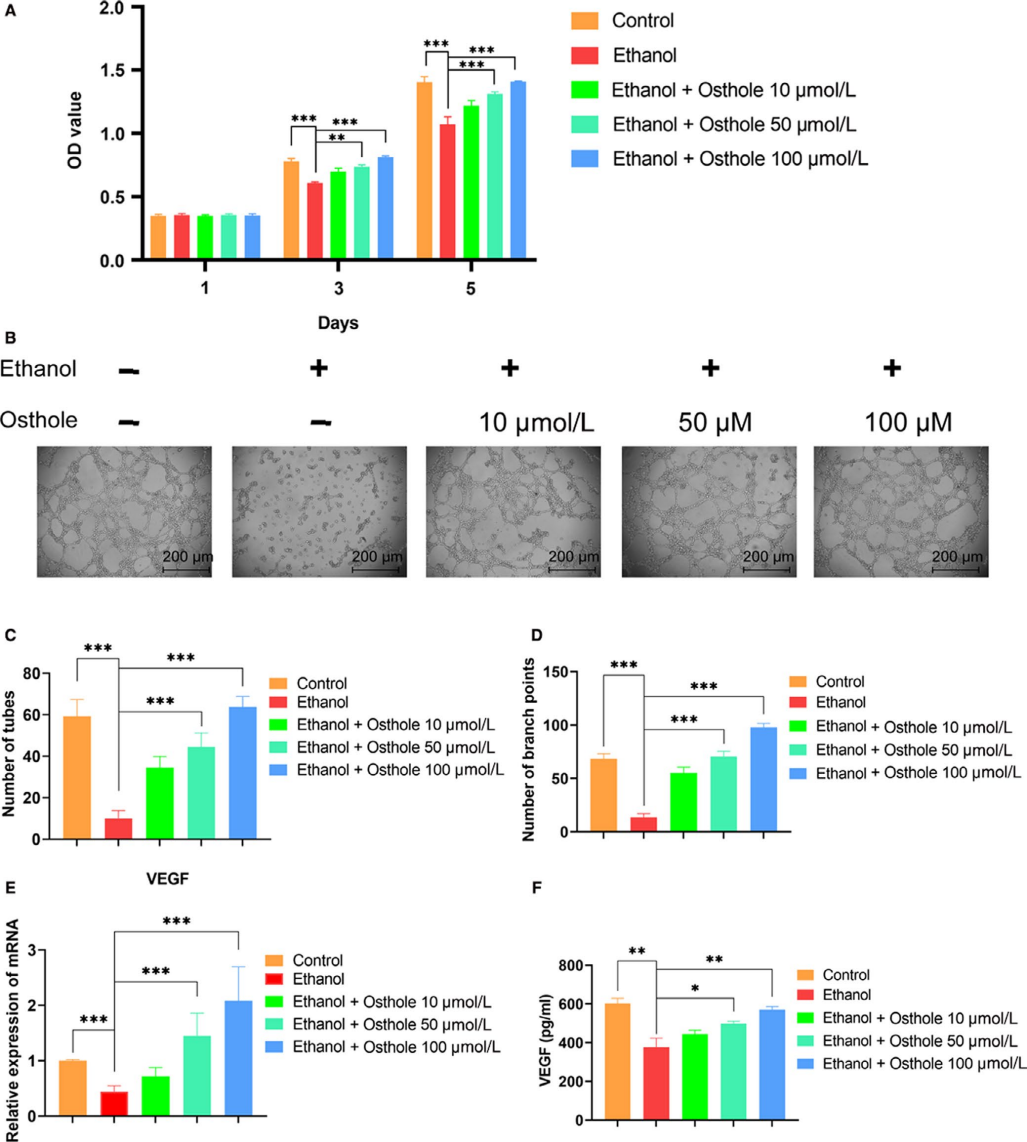
Osthole alleviated the ethanol‐induced inhibition on proliferation and vasculogenesis of HUVECs. A, Proliferation of HUVECs incubated in medium supplemented with ethanol and osthole as indicated by CCK 8 assay. Results were means ± SEM of four independent experiments in duplicate. B, The tube formation image of HUVECs. C,D, Quantitation of tubes and branch points. Results were means ± SEM of four independent experiments in duplicate. E,F, Both mRNA and protein levels of VEGF in HUVECs were decreased after 48 h incubation with ethanol but were substantially up‐regulated when supplemented with osthole. GAPDH was set as a normalization control. Results were means ± SEM of four independent experiments

CCK‐8 assay was employed to detect the proliferation of HUVECs. Ethanol could significantly inhibit the proliferation of HUVECs at day 3 and 5; however, osthole (10, 50, 100 μmol/L) was able to abolish the inhibitory effect of ethanol on HUVECs proliferation in a dose‐dependent manner (Figure 4A).

By tube formation assay, we found that much fewer loop structures formed when HUVECs were co‐cultured with ethanol for 12 hours; however, osthole could dose‐dependently counteract the detrimental effect of ethanol in the vasculogenesis of HUVECs (Figure 4B,C and D). VEGF is a crucial factor in vascular sprouting and maturation.^36^, ^37^ The RT‐PCR results indicated that the expression of VEGF was significantly decreased at 48 hours in the ethanol group. However, osthole could dose‐dependently reverse the inhibition caused by ethanol (Figure 4E). We also detected the level of VEGF by ELISA. VEGF was significantly decreased by ethanol and substantially rescued by osthole dose‐dependently (Figure 4F). In short, these data indicated that 50 mmol/L ethanol could impair tube formation in vitro whereas osthole could dose‐dependently restore vasculogenesis capacity of HUVECs.

### 3.5 Ethanol‐induced ONFH was alleviated by osthole in the rat model

**Figure 5:**
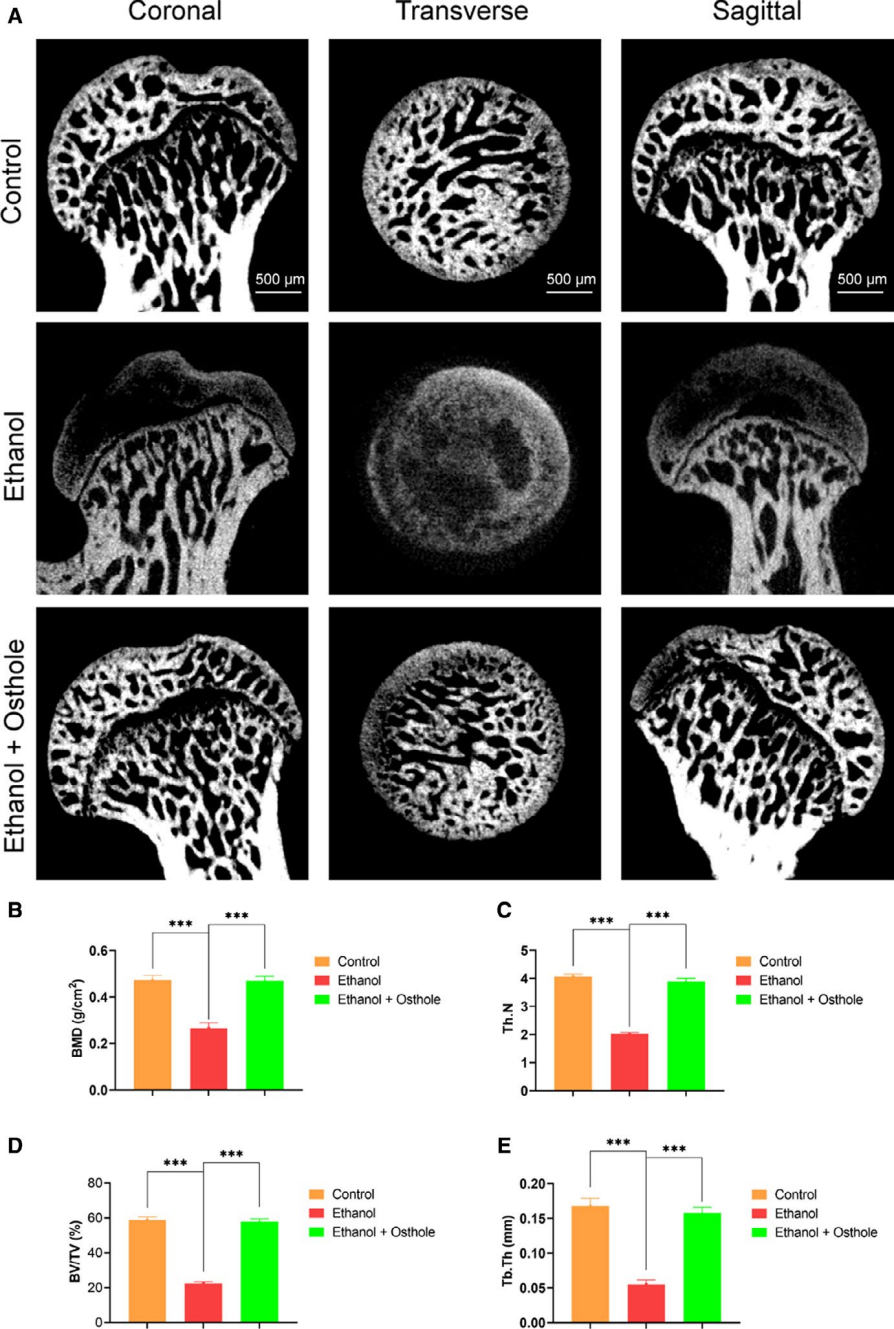
Micro‐CT scanning and analysis. A, Micro‐CT scanning images of the femoral head divided by group and section. Subchondral trabecular bone was dramatically impaired in the ethanol group; however, the trabecular structure was largely restored in the ethanol + osthole group. B‐E, BMD, Tb.N, BV/TV and Tb.Th were calculated based on reconstructed CT images. Results were means ± SEM of five specimen. BMD, bone mineral density; BV/TV, bone volume/tissue volume; Tb.N, trabecular number; Tb.Th, trabecular thickness

In order to determine the effect of osthole on ethanol‐induced ONFH, the rat model of ONFH was established via feeding Lieber‐DeCarli ethanol‐containing liquid diet for 6 weeks.^34^ Intraperitoneal injection of osthole or saline was set as the treatment or the control group. Firstly, micro‐CT scanning was used to evaluate the osseous structures of the femoral head. In total, 8 of 10 rats in the ethanol group had obvious and visible signs of osteonecrosis. However, only one rat in the ethanol + osthole group showed mild ONFH signs, and there was no osteonecrosis in the control group (Figure 5A). Quantitative analysis of the micro‐CT data was used to further clarify the harmful effect of ethanol and the protective role of osthole. Critical parameters, including BMD (0.27 ± 0.02 g/cm^3^), Tb.N (2.03 ± 0.05), BV/TV (22.43 ± 1.18%) and Tb.Th (0.06 ± 0.01 mm), in the ethanol group were remarkably decreased compared to the control group (0.47 ± 0.02 g/cm^3^, 4.07 ± 0.09, 59.03 ± 4.97%, 0.17 ± 0.01 mm, respectively). Notably, osthole could improve these parameters, reflecting its beneficial role in the restoration of bone homoeostasis (Figure 5B‐E). Animals in all groups had a gradual and similar weight gain during the experiment (Figure S2).

**Figure 6:**
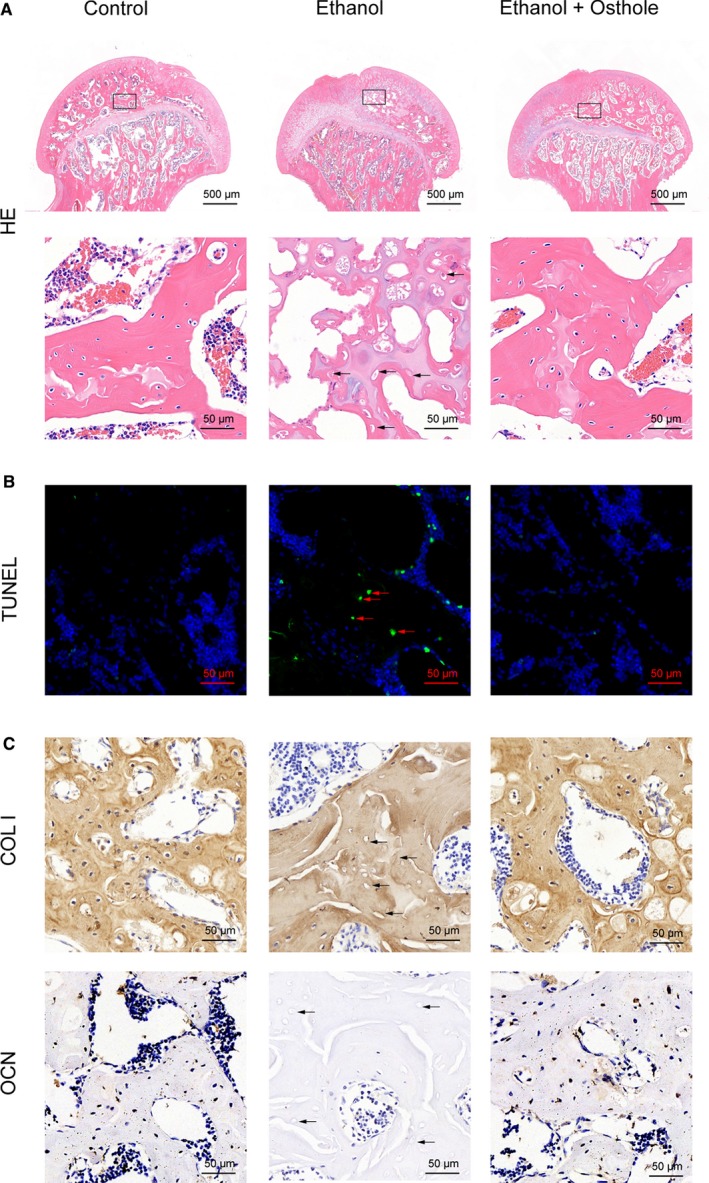
Osthole ameliorated ethanol‐induced ONFH in the rat model. A, H & E staining indicated obvious osteonecrosis in the ethanol group. Empty lacunae in the subchondral trabeculae (black arrows) were observed in the ethanol group. B, TUNEL showed increased apoptosis in the ethanol group, which was alleviated by osthole treatment. The TUNEL positive cells were indicated in the trabeculae of the femoral head (red arrows). C, Immunohistochemical staining of COL I and OCN. Fewer cells in the trabeculae (black arrows) were positive for COL I and OCN in the ethanol groups; however, the staining was substantially improved with osthole treatment

Next, the identification of hip osteonecrosis was confirmed by histopathological examinations, including H&E, TUNEL and immunohistochemical staining. The osteonecrosis is defined as diffuse empty lacunae or the presence of pyknotic nuclei in bone trabeculae accompanied by bone marrow cell necrosis.^38^ As shown in Figure 6A, there were diffuse empty lacunae and pyknotic nuclei in trabeculae with bone marrow haematopoietic cellular debris in medullary spaces in the ethanol group. On the contrary, the feature of osteonecrosis was dramatically alleviated in the ethanol + osthole group. Notably, there was no apparent histopathological abnormality in the control group. One of the vital pathological changes of ONFH development is apoptosis, as demonstrated by TUNEL staining in this study. There were significantly more positive staining cells in the trabeculae in the ethanol group; however, much fewer positive staining cells were observed in the ethanol + osthole group (Figure 6B). Finally, immunohistochemical staining of COL I and OCN was used to assess the effects of ethanol and osthole on bone formation. There was a weaker staining of COL I and OCN in the ethanol group, implying the decreased osteogenic activity. However, the inhibitory effect of ethanol could be neutralized by the co‐treatment of osthole (Figure 6C).

**Figure 7:**
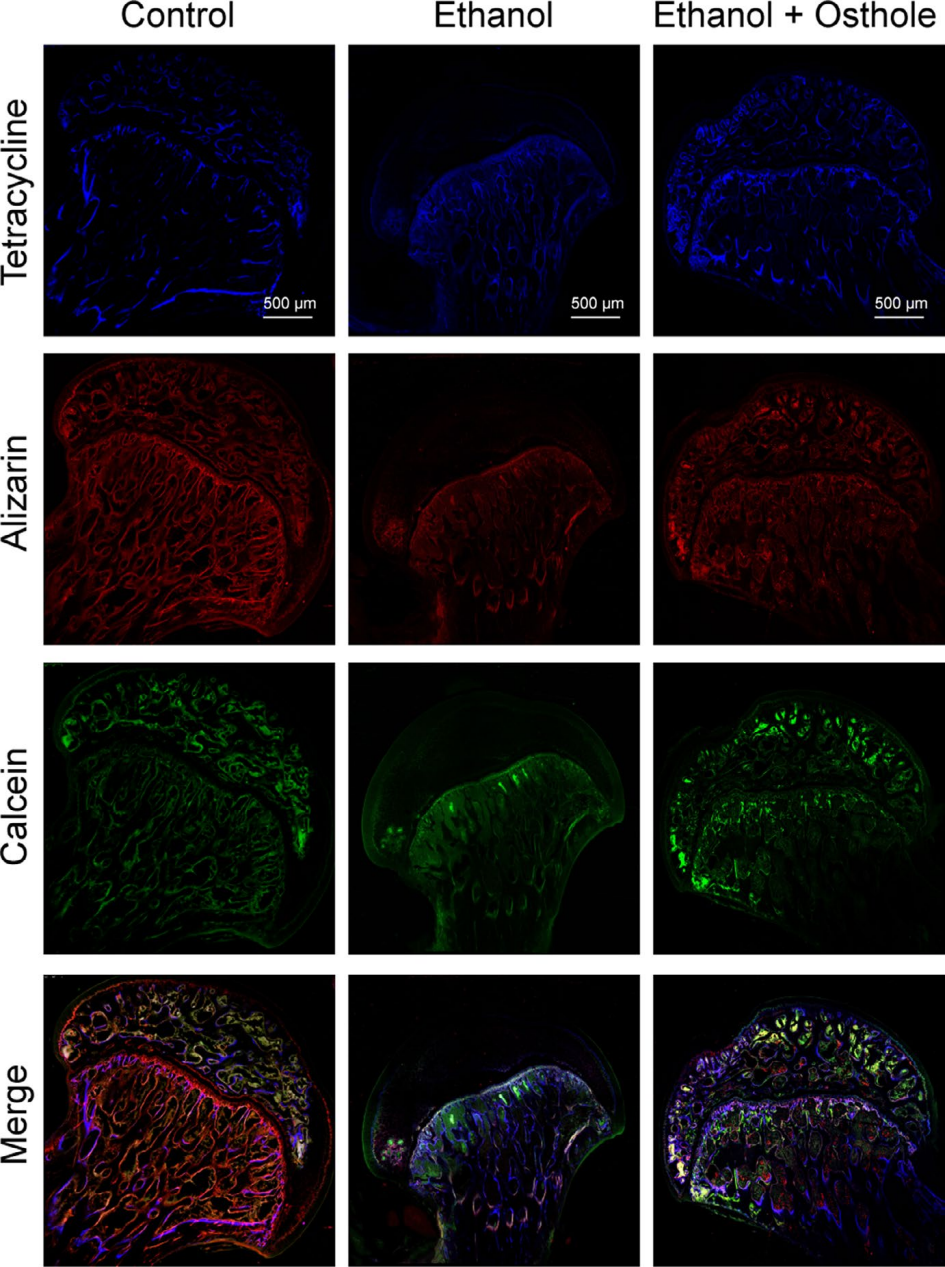
The protective effects of osthole against ethanol‐induced ONFH in the rat model. The fluorochrome labelling indicated significantly reduced new bone formation in the ethanol‐treated rat. Bone formation capacity was substantially restored with osthole treatment

Fluorochrome labelling with tetracycline, Alizarin red S and calcein‐AM was performed to monitor dynamic bone formation and mineralization in the femoral head. As shown in Figure 7, tetracycline (blue), Alizarin red S (red) and calcein (green) were deposited within a broader area of the subchondral trabeculae of the femoral head in the control group. On the contrary, there was a poor staining in the subchondral trabeculae for ethanol‐feeding rats, which implied the decreased capacity of new bone formation and impaired bone homoeostasis. Noteworthily, after osthole administration, the distribution of tetracycline, alizarin red and calcein was improved to a much better level, suggesting the beneficial effect of osthole in the prevention of ethanol‐induced ONFH.

## 4 DISCUSSION

Alcohol consumption is considered as a vital risk factor for non‐traumatic ONFH.^6^, ^11^, ^39^ A myriad of epidemiologic studies indicated that a substantial number of ONFH patients were due to alcohol overuse.^40^, ^41^ Prior studies showed an increased fat deposition and vascular pressure but decreased osteogenic activity in alcohol‐induced ONFH.^11^, ^42^ However, the natural history of ONFH determines the majority cases might undergo structural failure of the femoral head. There is a lack of standard protocol for the treatment of ONFH, implying the complexity of the recalcitrant disease. In this study, for the first time, osthole was demonstrated to rescue the ethanol‐induced ONFH in the rat model *via* stimulating bone formation, driving vascularization and inhibiting adipogenesis.

Firstly, we found that ethanol could significantly inhibit the proliferation and osteogenic differentiation capacity of BMSCs. The osteogenic‐related biomarkers, at both mRNA and protein levels, were dramatically inhibited by ethanol. Meanwhile, COL I and OCN immunofluorescence staining revealed that ethanol could impair bone formation. Wnt/β‐catenin pathway is one of the most important signals involved in the osteogenesis of BMSCs. Herein, we found that the level of β‐catenin was remarkably reduced by ethanol, in consistent with previous reports.^16^, ^43^, ^44^ Intriguingly, osthole could dose‐dependently counteract the inhibition of ethanol on the proliferation and osteogenic differentiation of BMSCs. In addition, osthole could dose‐dependently abolish the inhibition of ethanol on β‐catenin expression to augment osteogenesis, in line with previous reports.^29^, ^45^ In vivo, typical pathological changes of hip osteonecrosis were observed by micro‐CT scanning and H & E staining in the ethanol group, whereas only mild osteonecrosis was presented in the animal with supplementary osthole administration. There were more apoptotic cells in the ethanol group, reflecting apoptosis was an essential phenomenon in the development of ONFH. However, osthole might play an antiapoptotic role to ameliorate stress‐induced tissue injury.^46^

Intramedullary fat deposition is revealed as a critical pathological feature in the progress of ONFH, characterized by enlarged cellular size and increased number of adipocytes.^47^, ^48^, ^49^ The strengthened capacity of adipogenesis in ethanol‐induced ONFH has been revealed in several animal models.^50^, ^51^, ^52^ Notably, promoted adipogenic differentiation led to disordered osteogenic differentiation, and the accumulation of adipocytes might lead to a high intramedullary pressure and impair brittle blood circulation in the femoral heads.^50^ Moreover, fat emboli in vessels and sinusoids can also indirectly induce intravascular coagulation *via* the complement signalling pathways and deposition of immune complex, whereas antithrombotic reagents have protective effects to promote circulation and ameliorate ONFH.^53^, ^54^ In this work, we found that ethanol could stimulate BMSCs towards adipogenic differentiation. However, it was demonstrated that osthole could augment osteogenesis and retard adipogenesis in the scenario of ethanol administration. The result is consistent with the previous report that osthole could prevent alcohol‐induced fatty liver.^19^ Therefore, the beneficial effect of osthole on ethanol‐induced ONFH might function via inhibiting the formation of adipocytes, decreasing the number of fat emboli and improving perfusion in the femoral heads.

Currently, it is recognized that ONFH is not merely a kind of skeletal disease, but involves a series of systemic pathologies, especially intraosseous circulation.^55^, ^56^, ^57^ ONFH might be considered as a vascular disease with attenuated blood flow and a specific bone disease with changed marrow cell differentiation and function.^2^ In addition, previous reports indicated that high dose of alcohol exerted a detrimental effect on angiogenesis by regulating the expression of angiogenic‐related genes of VEGF, hypoxia‐inducible factor (HIF) and fibroblast growth factor (FGF).^13^, ^14^ Notably, VEGF has a vital role in the repair process for bone regeneration.^58^ In our study, we found that 50 mmol/L ethanol treatment could significantly inhibit the proliferation and tube formation of HUVECs. However, osthole could rescue the inhibitory effect of ethanol in a dose‐dependent manner and substantially restore the function of tube formation of HUVECs. Moreover, we demonstrated that osthole could dose‐dependently reverse the inhibition effect of ethanol on VEGF expression. The beneficial role of osthole in driving vascularization was of great importance for the prevention of ethanol‐induced ONFH.

## 5 CONCLUSIONS

Taken together, for the first time, osthole was demonstrated to prevent ethanol‐induced ONFH and ameliorate radiological and histological features of the femoral head in vivo. Osthole could stimulate bone formation, drive vascularization and retard adipogenesis. Hence, osthole may become a potential pharmacotherapeutic method for the treatment of ONFH.

## CONFLICT OF INTEREST

The authors confirm that there are no conflicts of interest.

## Supporting information

Figure S1

Figure S2

Table S1

## Data Availability

The data that support the findings of this study are available from the corresponding author upon reasonable request.
