# Supplementary figures and images for: Osthole stimulates bone formation, drives vascularization and retards adipogenesis to alleviate alcohol‐induced osteonecrosis of the femoral head
Source: J Cell Mol Med. 2020 Mar 5;24(8):4439–51. doi: 10.1111/jcmm.15103 (PMC7176840; doi:10.1111/jcmm.15103)

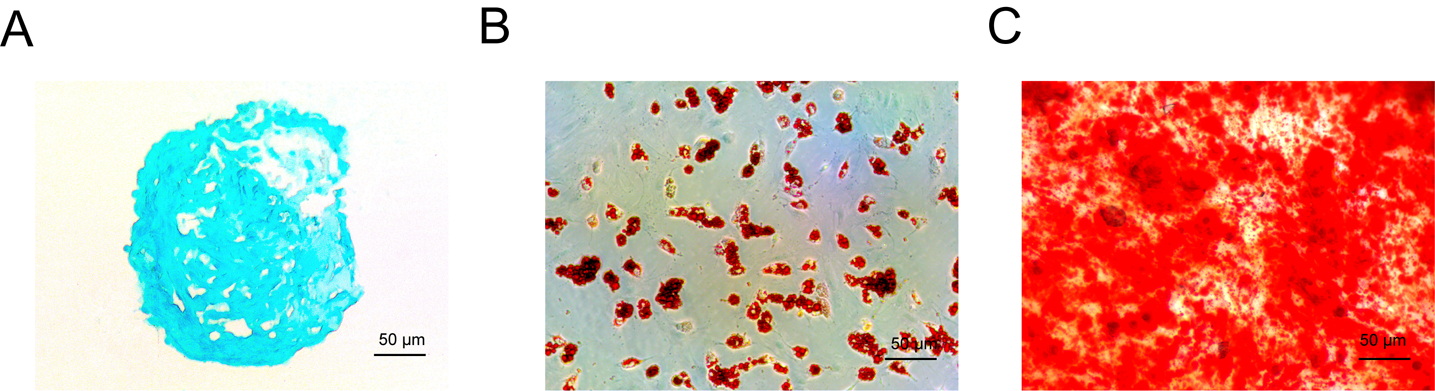

Supplement: Supplementary file 1 — Figure S1 [file JCMM-24-4439-s001.tif]

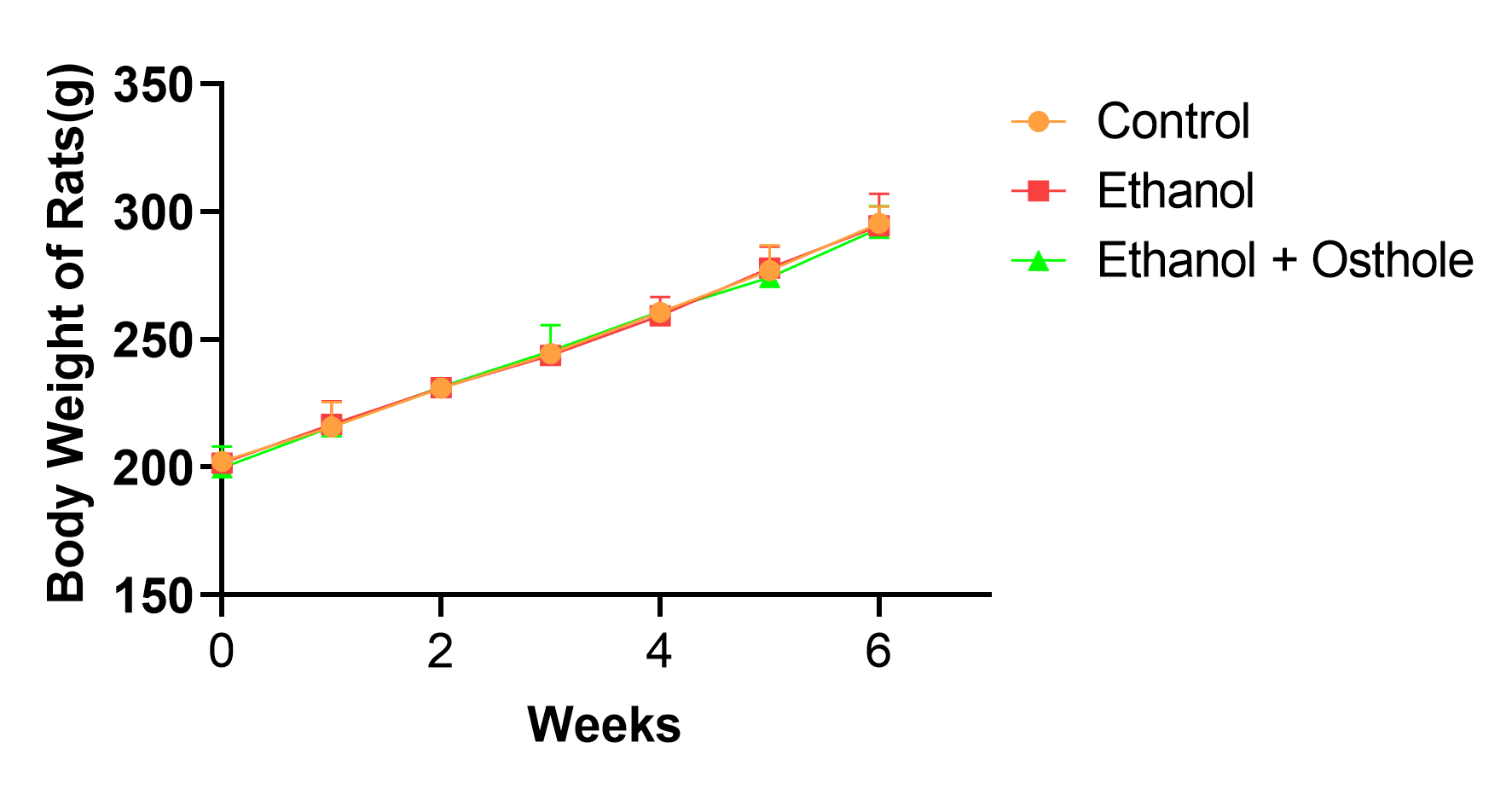

Supplement: Supplementary file 2 — Figure S2 [file JCMM-24-4439-s002.tif]
