# Supplementary material for: Osthole stimulates bone formation, drives vascularization and retards adipogenesis to alleviate alcohol‐induced osteonecrosis of the femoral head
Source: J Cell Mol Med. 2020 Mar 5;24(8):4439–51. doi: 10.1111/jcmm.15103 (PMC7176840; doi:10.1111/jcmm.15103)
Supplement: Supplementary file 3 — Table S1 [file JCMM-24-4439-s003.docx]

**Supplementary Table 1**. The RT-PCR primers used in this study

| Gene | Forward sequence (5´-3´) | Reverse sequence (5´-3´) |
| --- | --- | --- |
| GAPDH | CAGGTTGTCTCCTGCGACTT | TATGGGGGTCTGGGATGGAA |
| COL I | CTCAAGAAGTCCCTGCTCCTC | GACTGTCTTGCCCCAAGTTC |
| OCN | GCATCCTTGGCTTTGCAGTC | AGTGTTTGCTGTAATGCGCC |
| OPN | CCGTTTAGGGCATGTGTTGC | CCGTCCATACTTTCGAGGCA |
| VEGF | CTGGGCTGTTCTCGCTT | CCCCTCTCCTCTTCCTTCT |
| PPARγ | CCGCATTTTTCAAGGGTGCC | CCGCAGGCTTTTGAGGAACT |
| Leptin | CTGAGTGCTGGGAGCTTGAT | GACGTGTGACAGAGGTACGG |
